# Case Report: Severe ARDS in a Pediatric Hematopoietic Stem-Cell Transplantation Recipient Caused by Disseminated Toxoplasmosis

**DOI:** 10.3389/fped.2021.810718

**Published:** 2022-01-26

**Authors:** Sara de la Mata Navazo, María Slöcker Barrio, Marina García-Morín, Cristina Beléndez, Laura Escobar Fernández, Elena María Rincón-López, David Aguilera Alonso, Jesús Guinea, Mercedes Marín, Laura Butragueño-Laiseca, Jesús López-Herce Cid

**Affiliations:** ^1^Pediatric Intensive Care Department, Hospital General Universitario Gregorio Marañón, Madrid, Spain; ^2^Instituto de Investigación Sanitaria Gregorio Marañón, Hospital General Universitario Gregorio Marañón, Madrid, Spain; ^3^Research Network on Maternal and Child Health and Development (RedSAMID), Hospital Universitario Gregorio Marañón, Madrid, Spain; ^4^Department of Maternal and Child Public Health, School of Medicine, Complutense University of Madrid, Madrid, Spain; ^5^Pediatric Hematology and Oncology Unit, Department of Pediatrics, Hospital General Universitario Gregorio Marañón, Madrid, Spain; ^6^Pediatric Infectious Diseases Unit, Department of Pediatrics, Hospital General Universitario Gregorio Marañón, Madrid, Spain; ^7^Clinical Microbiology and Infectious Diseases Department, Hospital General Universitario Gregorio Marañón, Madrid, Spain

**Keywords:** toxoplasma and toxoplasmosis, hematopoietic stem cell transplantation (HCST), leukemia, ARDS, pediatric, ventilation, intensive care unit

## Abstract

*Toxoplasma gondii* infection is a severe complication of hematopoietic stem-cell transplantation (HSCT) recipients that can remain unnoticed without a high clinical suspicion. We present the case of a 6-year-old patient with acute lymphoblastic leukemia and HSCT recipient who was admitted to the Pediatric Intensive Care Unit (PICU) on post-transplantation day +39 with fever, hypotension, severe respiratory distress and appearance of a lumbar subcutaneous node. She developed severe Acute Respiratory Distress Syndrome (ARDS) and underwent endotracheal intubation and early mechanical ventilation. Subsequently, she required prone ventilation, inhaled nitric oxide therapy and high-frequency oscillatory ventilation (HFOV). An etiologic study was performed, being blood, urine, bronchoalveolar lavage and biopsy of the subcutaneous node positive for *Toxoplasma gondii* by Polymerase Chain Reaction (PCR). Diagnosis of disseminated toxoplasmosis was established and treatment with pyrimethamine, sulfadiazine and folinic acid started. The patient showed clinical improvement, allowing weaning of mechanical ventilation and transfer to the hospitalization ward after 40 days in the PICU. It is important to consider toxoplasmosis infection in immunocompromised patients with sepsis and, in cases of severe respiratory distress, early mechanical ventilation should be started using the open lung approach. In *Toxoplasma* IgG positive patients, close monitoring and appropriate anti-infectious prophylaxis is needed after HSCT.

## Background

Acute Respiratory Distress Syndrome (ARDS) is an interstitial lung disease secondary to alveolar-capillary barrier disruption. It may be caused by primary pulmonary conditions such as severe pneumonia, or in severe systemic inflammatory settings like pancreatitis, polytrauma or massive transfusions, among other causes (1).

The incidence of *Toxoplasma gondii* infection in hematopoietic stem-cell transplantation (HSCT) recipients is 0.4-9%, and it is strongly related to severe immunosuppression. The most frequent clinical expression of *Toxoplasma gondii* infection in immunocompromised patients is cerebral toxoplasmosis, followed by pulmonary disease progressed into ARDS and disseminated toxoplasmosis. However, given the low specify of symptoms, definitive diagnosis of *Toxoplasma* infection is occasionally established after a post-mortem necropsy (2).

Multiple cases of disseminated toxoplasmosis have been reported in adult patients; however, the evidence available about this infection in pediatric HSCT recipients is scarce and reflects elevated morbidity and mortality rates. We describe a rare case of toxoplasmosis with a favorable outcome in a critically ill pediatric patient. This case report may contribute to better understand this rare complication in pediatric critical care patients. It is essential that toxoplasma infection is considered in HSCT patients presenting with fever and multisystemic involvement, especially if they come from areas with a high seroprevalence of *Toxoplasma* (2–5).

This study was approved by the Local Ethics Committee. Parents gave written informed consent for the publication of this case report in accordance with the Declaration of Helsinki.

## Case Report

We present the case of a 6-year-old patient from Honduras who was diagnosed with preB acute lymphoblastic leukemia at 18 months of age, who had two intramedullary relapses in her home country. In our hospital, she received relapse treatment with dexamethasone, mitoxantrone, vincristine, peg-asparaginase, bortezomib and intrathecal chemotherapy. After the induction phase, 85% of blast cells in bone marrow were observed, entering a chemo refractory situation. Second-line treatment with inotuzumab was administered, reaching complete remission, and an allogenic HSCT was performed, being her father the donor. Positive IgG and negative IgM were detected in the patient's serological screening for *Toxoplasma gondii* conducted prior to transplantation, whereas the donor had negative IgG results. After stem-cell infusion, prophylactic treatment with micafungin and levofloxacin was started. She had received trimethoprim-sulfametoxazole (TMS-SMX) from day−8 until day −2 before cell infusion, which was discontinued during engraftment. As an early complication, cytomegalovirus (CMV) reactivation was detected on day + 24 and treatment with valganciclovir was initiated.

**Figure 1:**
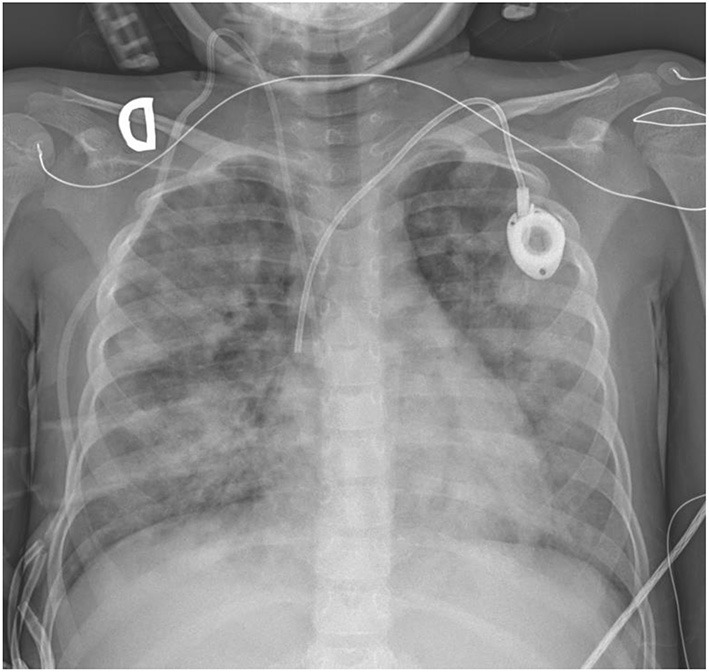
Chest radiography on admission.

On post-transplantation day + 35, the patient presented with fever, cough, diarrhea and appearance of a subcutaneous node in the lumbar area. Suspecting sepsis in a neutropenic patient, empiric broad-spectrum treatment with meropenem, teicoplanin and ganciclovir was started. Her status worsened and she developed hypoxemic respiratory failure and hypotension, requiring admission to the Pediatric Intensive Care Unit (PICU). A chest-X ray was performed, revealing diffuse heterogeneous bilateral infiltrates (Figure 1). Blood test showed a total white blood cell (WBC) count of 22,200 cells per microliter, 16,200 neutrophils, a C-reactive protein (CRP) of 29 mg/dl and a procalcitonin of 47 mcg/l. Infectious ARDS was suspected, and anti-infectious treatment was increased, adding levofloxacin and amphotericin B. Intravenous TMS-SMX was initiated to treat a potential *Pneumocystis jirovecii* pneumonia.

**Figure 2:**
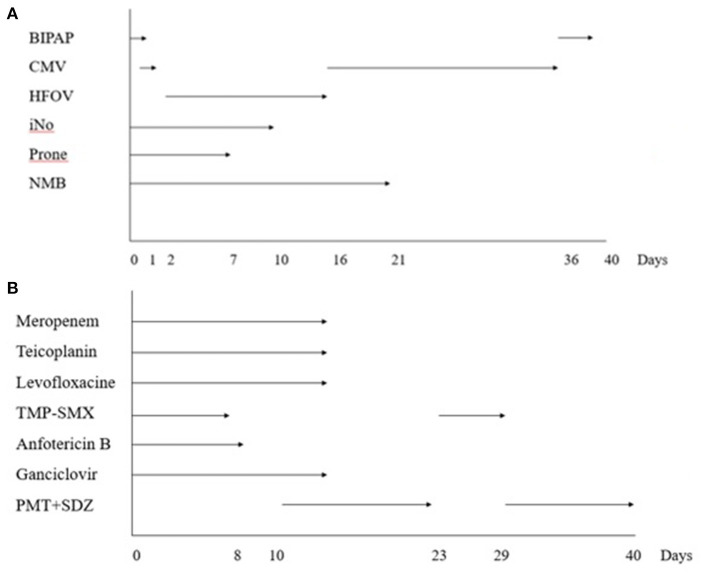
**(A)** Ventilatory support diagram. CMV, conventional mechanical ventilation; HFOV, high flow oscillatory ventilation; iNO, inhaled nitric oxide; NMB, neuromuscular blockade. **(B)** Anti-infectious treatment diagram. TMS-SMX, trimethoprim-sulfamethoxazole; PMT + SDZ, pyrimethamine + sulphadiazine.

Respiratory support with double level non-invasive mechanical ventilation (BIPAP) was started, but after 3 h she required endotracheal due to progressive respiratory failure. Management of ARDS required low tidal volume (4-6 ml/kg), PEEP up to 15 cm H_2_O and deep analgosedation and neuromuscular blockade. Transthoracic echocardiography on admission showed no abnormalities. Moreover, because of persistent hypoxemia with PaO_2_/FiO_2_ of 60 and an oxygenation index of 52, she was placed in prone position and therapy with inhaled nitric oxide (iNO) was started, initially at 20 parts per million (ppm) and subsequently decreased to a minimum effective dose of 10 ppm. Severe refractory hypoxemia persisted despite these measures; therefore, high-frequency oscillatory ventilation (HFOV) was initiated on the second day after admission and maintained for 16 days. Maximum ventilatory parameters included mean airway pressure 33 cm H_2_O, amplitude 68 cm H_2_O, frequency 5.5 Hz and FiO2 100%. Figure 2A shows the evolution of respiratory support.

**Figure 3:**
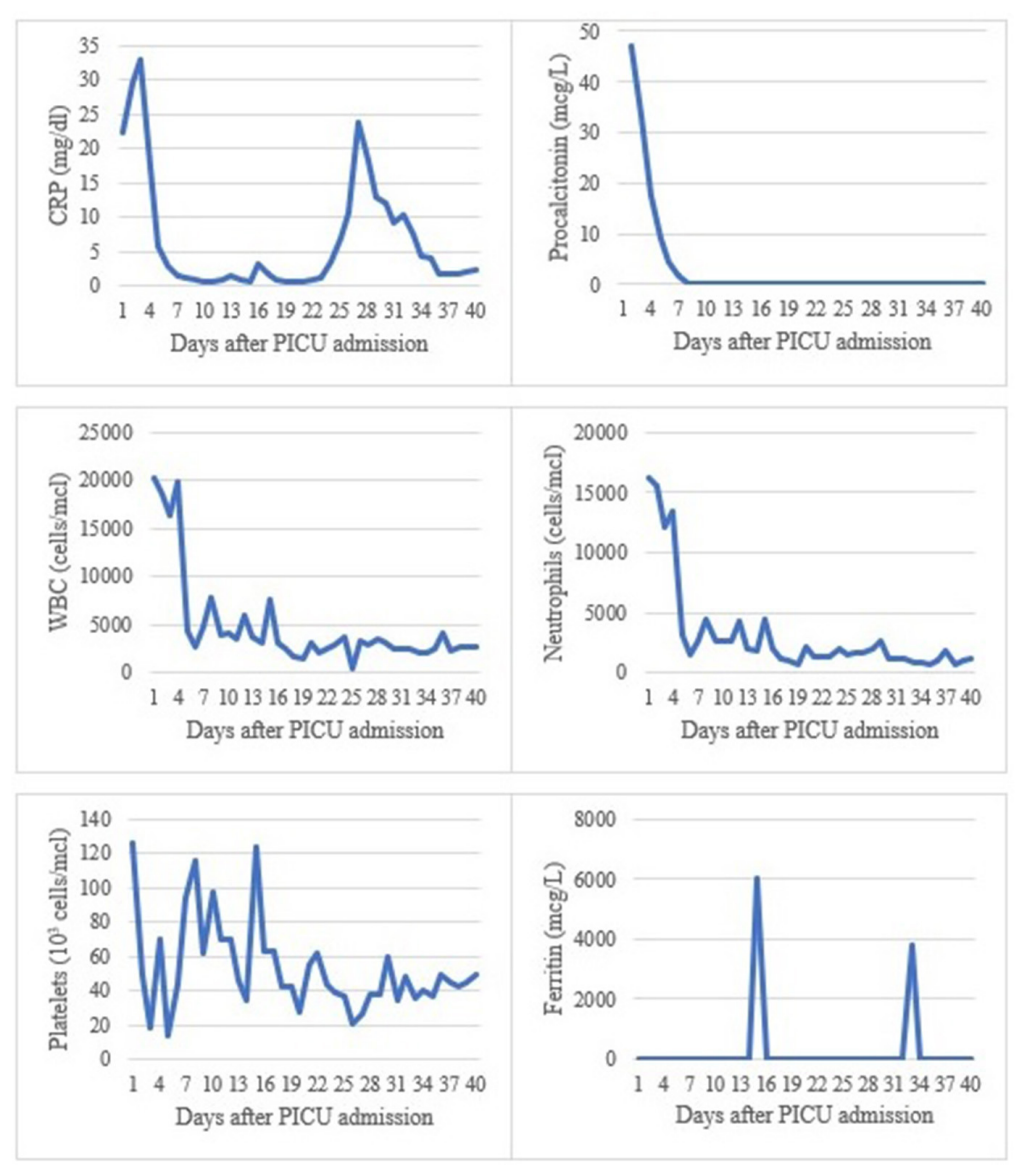
Summary of blood analytical values during PICU stay. CRP, C reactive protein; WBC, white blood cell; PICU, pediatric intensive care unit.

A comprehensive etiological study was performed on admission, obtaining a preliminary positive panfungal PCR in both bronchoalveolar lavage (BAL) and lumbar node biopsy after 9 days. Twenty-four hours later, targeted PCR confirmed the presence of *Toxoplasma gondii* in blood, urine, BAL and lumbar node tissue, which was consistent with disseminated toxoplasmosis, while fungal infection was discarded. Microscopic exam of the lumbar node tissue showed patched tissue necrosis, inflammatory cells and absence of tachyzoites. Treatment with pyrimethamine, sulphadiazine and folinic acid was started, and as subsequent microbiological tests were negative, the other antimicrobial therapies were progressively withdrawn (Figure 2B). After 13 days of anti-toxoplasma treatment, sulphadiazine crystals were detected in patient's urine and therapy with sulphadiazine and pyrimethamine had to be switched to intravenous trimethoprim-sulfamethoxazole. Urine sediment was normalized and, 6 days later, the previous treatment was resumed. Evolution of blood test values during PICU stay is shown in Figure 3.

After goal-directed treatment was started, patient's status improved and, 8 days later, ventilation was switched from HFOV to conventional mechanical ventilation. Neuromuscular blockade was interrupted on the 21st day after admission and, finally, she was extubated 36 days after PICU admission. A cerebral computed tomography scan excluded cerebral lesions consistent with toxoplasmosis, and neurological examination was normal after withdrawal of analgosedation.

Forty days after intensive care admission, the patient was transferred to the hospitalization ward, where she completed a 6-week treatment with pyrimethamine and sulphadiazine and, afterwards, prophylactic therapy with TMS-SMX was established. Serial PCR tests were negative and pulmonary function improved. The patient was discharged on post-transplantation day +90.

## Discussion

We present the case of a HCST recipient, born in a region of the world with high seroprevalence of *Toxoplasma*, and with a long course of immunosuppressive treatment, that developed a disseminated toxoplasmosis due to reactivation on the day +35 after transplantation, consisting of pulmonary disease with severe ARDS development and skin lesions. ARDS was managed according to pediatric guidelines, and toxoplasmosis was diagnosed with a positive PCR in BAL, blood, urine and skin tissue. She received targeted treatment with pirimetamin, sulphadiazine and folinic acid, that was temporarily switched to TMS-SMX because of crystalluria.

*Toxoplasma gondii* is an intracellular parasite that causes mild or even asymptomatic disease in immunocompetent hosts, with symptoms similar to those of flu or common cold. It is estimated to affect up to a third of the global population, with a higher seroprevalence in South America, tropical Africa or Eastern Europe (4).

In immunocompromised hosts, however, toxoplasmosis is a devastating infection, especially in allogenic HSCT recipients, caused by the reactivation of a previously acquired latent infection. The incidence of post-transplant toxoplasmosis is around 0.4-9%, and it usually develops within the first 6 months after infusion, with a higher incidence in the second and third month; although in our case, reactivation occurred earlier (day + 35). Martino et al. reviewed 4,231 HSCT recipients and observed 41 cases of toxoplasmosis (0.96%), showing a low incidence of this complication (5). However, there is a higher incidence in patients coming from high seroprevalence countries and those who are subject to high-intensity immunosuppressive regimens due to multiple relapses, like this patient (6). Other risk factors include cord blood transplantation, antithymocyte globulin administration, acute graft vs. host (AGVH) disease, CMV reactivation and absence of prophylactic treatment with TMS-SMX. Our patient met the two latter criteria (2, 6). In addition, most reported cases of toxoplasmosis are observed in patients whose donor was seronegative for *T. gondii* (7, 8).

Cases of pediatric HSCT toxoplasmosis most commonly exhibit central nervous system (CNS) involvement. However, pneumonitis and disseminated toxoplasmosis are less frequently detected, due to the unspecificity of symptoms and low awareness of the disease; as a result, they are generally found in post-mortem studies. Cases of immune dysregulation with hyperferritinemic sepsis secondary to toxoplasma infection have also been described, with fatal outcomes (9). Komitopoulou et al. reviewed 17 cases of toxoplasmosis in pediatric HSCT patients described in the literature since year 2000, with mortality rates of 52%, reaching 100% in patients with disseminated toxoplasmosis (2). Rauwolf et al. also reviewed six publications regarding toxoplasmosis in pediatric HSCT receptors, describing 13 toxoplasmosis cases, most of them with CNS involvement (6). Other authors establish a global mortality rate of post-transplant toxoplasmosis of 50% (5, 10).

Our patient developed pulmonary, systemic and cutaneous involvement, and despite having disseminated toxoplasmosis, imaging tests did not show cerebral lesions consistent with toxoplasmosis, and neurological examination was normal.

Regarding ARDS development, this disease may be triggered by numerous causes, such as sepsis, pneumonia, pulmonary contusion, drowning, polytrauma, massive transfusion, and, generally, high systemic inflammatory situations (1, 11). *Toxoplasma* pneumonitis may cause a release of inflammatory mediators, that can accumulate in the alveoli and microcirculation of the lung, damaging alveolar epithelium and vascular endothelium. This leads to edema, decreased lung compliance and a decrease in gas exchange (12).

Pediatric ARDS (PARDS) definitions, diagnostic and therapeutic consensus were established in the 2014 Pediatric Acute Lung Consensus Conference (PALICC) (1, 11). Our patient was managed according to these guidelines, based on the “open lung strategy,” with administration of a low tidal volume (3-6 ml/kg) and an elevated PEEP (usually > 10-15 cm H_2_O), to avoid alveolar collapse and increase pulmonary distensibility, associating alveolar recruitment maneuvers (11–14).

In our case, the initial severity and persistence of hypoxemia despite optimized conventional mechanical ventilation, sedation and neuromuscular blockade eventually required prone ventilation, use of iNO and HFOV. These measures are not recommended as routine practice, although they can be considered in patients with severe PARDS (1, 15, 16). Extracorporeal membrane oxygenation (ECMO) could be considered in patients with severe PARDS and potentially reversible respiratory failure or pulmonary transplant candidates (1, 11, 13). In this patient, ECMO support was discussed as a rescue therapy in case of persistence of refractory hypoxemia, but finally was not necessary due to patient's improvement.

Although there is no clear consensus concerning initiation and duration of *T. gondii* prophylaxis, many HSCT guidelines recommend starting anti-*Toxoplasma* prophylaxis as soon as feasible after HSCT, but no later after engraftment is established (usually from day + 30) (7). As stated in ESCMID guidelines, the start of prophylaxis right after HSCT may expose the patient to some degree of toxicity and delayed engraftment, whereas postponing prophylaxis places the patient at risk for toxoplasmosis (8). The most frequently used drug is trimethoprim-sulfamethoxazole, administered either daily or three times per week (4, 9, 17, 18), although some authors suggest the prophylactic use of atovaquone as an alternative if engraftment is delayed and TMP/SMX cannot be used due to myelosuppression (7). If delayed prophylaxis is preferred, it is also recommended to perform routine blood PCR screening in patients with a positive pre-transplant serology (8, 9, 18).

In our case, TMS-SMX prophylaxis was interrupted during the engraftment phase, according to transplant guidelines (4, 17–19), due to its potential myelotoxicity, and it had not been restarted by the onset of symptoms, as our protocol at that time was to wait until day +35 after transplant to restart TMS-SMX. Retrospectively, we may have missed an opportunity to introduce *Toxoplasma* prophylaxis because, although the patient needed periodic platelet transfusions by the time of PICU admission, neutrophile engraftment had already occurred on day +17. Besides, routine *Toxoplasma* PCR testing was not included in our local HSCT follow-up protocol at that time, which would have facilitated early detection of the infection. In our center, this case motivated a multidisciplinary review of the management of *Toxoplasma* IgG+ patients, including weekly *Toxoplasma* PCR blood screening during the engraftment phase and prophylaxis with TMS-SMX as soon as engraftment has occurred and up to day +180.

First-line treatment for toxoplasma includes pyrimethamine plus sulphadiazine, associated to adjuvant folinic acid to avoid pyrimethamine's hematological toxicity, for 4-6 weeks. Other second-line treatments are pyrimethamine plus clindamycin or trimethoprim-sulfamethoxazole (19). Some studies suggest that quinolone-derived drugs, like levofloxacin, may also have potential anti-*Toxoplasma* activity (15, 16).

Our patient had a favorable ICU course despite severe illness. This could be partially attributed to the early start of TMS-SMX and levofloxacin as part of the empyrical broad-spectrum therapy to treat a potential *Pneumocystis jirovecii* pneumonia, which also proved to be effective against Toxoplasma. Consequently, the patient received unintentionally targeted treatment in the first days after ICU admission, which probably led to clinical improvement.

The fact that panfungal PCR was initially positive can be explained because the primers used in fungal sequencing can cross-react with several protozoa. In the review performed by Gomez et al. (20), D2 sequencing in 117 patients diagnosed 5 cases of invasive protozoal infections due to Toxoplasma gondii (*n* = 3), Trypanosoma cruzi, and Leishmania spp. In our case, although the initial suspicion was an invasive fungal infection, *Toxoplasma* was confirmed 24 h later, leading to the start of appropriate antiparasitic therapy.

Sulphadiazine crystalluria is a rare complication, although it has been described in patients receiving sulphamide-derived drugs, especially in HIV patients with toxoplasma encephalitis (21, 22), that may associate renal impairment due to sulphadiazine in 1.9-7.5% of cases. These crystals can even obstruct the urinary tract and, consequently, produce obstructive renal failure. Therefore, it is recommended to start hyperhydration and switch treatment to trimethoprim-sulfamethoxazole or pyrimethamine plus clindamycin. In our case, this complication was detected early when macroscopically visible crystals were detected inside the urinary catheter. After treatment modification, urine sediments were periodically analyzed until normalization. Pyrimethamine and lower-dose sulphadiazine were resumed later.

## Conclusion

*Toxoplasma* infection must be considered in differential diagnosis of immunocompromised patients with fever, multisystemic involvement and respiratory distress. Patients' prognosis benefits from early initiation of targeted treatment.

Prior to HSCT, seropositive patients should be routinely screened for *Toxoplasma* with weekly PCR tests to ensure early detection of parasite reactivation. Infectious prophylaxis should be started as soon as engraftment is established.

## Data Availability Statement

The original contributions presented in the study are included in the article, further inquiries can be directed to the corresponding author/s.

## Ethics Statement

Written informed consent was obtained from the minor(s)' legal guardian/next of kin for the publication of any potentially identifiable images or data included in this article.

## Author Contributions

SM and MS contributed to conception and design of the study. SM drafted the first draft of the manuscript. MS, MG-M, DA, and ER-L wrote sections of the manuscript. MG-M, LE, CB, ER-L, DA, MM, and LB-L thoroughly reviewed previous evidence and performed bibliographic search. MM and JG performed the microbiological tests needed for the patient's diagnosis. JL-H coordinated article writing and submission. All authors contributed to manuscript revision, read, and approved the submitted version.

## Conflict of Interest

The authors declare that the research was conducted in the absence of any commercial or financial relationships that could be construed as a potential conflict of interest.

## Publisher's Note

All claims expressed in this article are solely those of the authors and do not necessarily represent those of their affiliated organizations, or those of the publisher, the editors and the reviewers. Any product that may be evaluated in this article, or claim that may be made by its manufacturer, is not guaranteed or endorsed by the publisher.
